# Molecular and histopathological confirmation of clinically diagnosed lumpy skin disease in cattle, Baghdad Province of Iraq

**DOI:** 10.14202/vetworld.2019.1826-1832

**Published:** 2019-11-23

**Authors:** Hasanain A. J. Gharban, Sattar J. J. Al-Shaeli, Hams H. H. Al-Fattli, Muthanna N. K. Altaee

**Affiliations:** 1 Department of Internal and Preventive Medicine, College of Veterinary Medicine, Wasit University, Wasit, Iraq; 2 Department of Histology and Anatomy, College of Veterinary Medicine, Wasit University, Wasit, Iraq; 3 Department of Clinical Laboratory Science, College of Pharmacology, University of Al-Qadisiyah, Al-Qadisiyah, Iraq; 4 Private Clinic, Wasit, Iraq

**Keywords:** cattle, histopathology, Iraq, lumpy skin disease, polymerase chain reaction

## Abstract

**Aim:**

This study aimed to confirm the clinically diagnosed cattle with lumpy skin disease (LSD) at Baghdad Province/Iraq from October 2018 to March 2019.

**Materials and Methods:**

Molecular polymerase chain reaction (PCR) and histopathology were applied for the detection of LSD among 71 infected cattle issued for slaughter.

**Results:**

Pre-slaughter clinical examination showed significant increases (p<0.05) in values of temperature (39.7±0.74°C), pulse (96.42±3.51), and respiratory (33.54±0.63) rates. Enlargement of lymph nodes (prescapular, supramammary, and prefemoral), lacrimation, mucopurulent nasal discharge, salivation, edema in limbs and head among severe infected cases, and marked fall in milk production was seen. An association of LSD to risk factors (age, gender, and areas) showed that there is significant elevation in prevalence of disease in >2-5 years (54.93%) rather than other age groups (>5 and <2 years)in females (73.24%) than males (26.76%); and in sub-rural (42.25%) and rural (39.44%) compared to urban (18.31%) areas. Postmortem examination appeared nodular lesions in upper parts of the digestive system (9.86%), rumen (2.82%), upper respiratory tracts (7.04%), and lung (4.23%). The PCR examination of *P32* and thymidine kinase antigenic genes showed 90.14% and 60.56% positive samples, respectively. Histopathological analysis of nodular skin biopsies showed edema, hyperemia, acanthosis, severe hydropic degeneration, and hyperkeratosis in epidermis; whereas, mononuclear cell infiltration, inclusion bodies, and vasculitis seen in the dermis.

**Conclusion:**

PCR and histopathology assay could be a potential method to confirm the LSD infection concomitant with clinical examination.

## Introduction

Lumpy skin disease (LSD) is an acute to chronic bovine viral disease caused by LSD virus (LSDV) that classified under Capripox genus of *Poxviridae* family [1]. Initially, the disease that first reported in 1929 in Zambia is suspected to be resulted by either poisoning or hypersensitivity to an insect bite, until the infectious nature was recognized in 1943 [2,3]. Since then, LSD is spread to other southern and northern countries of Africa, and the Middle East incurred enormous economic losses in cattle livestock [4]. In Iraq, the first infected foci of LSD were indicated in many regions of Nineveh and Baghdad Provinces in 2013 [5]. However, the causative pathogen transmits mainly by biting insects such as mosquitoes and biting flies which act as vectors and less commonly by direct contact with skin lesions, milk, nasal discharge, saliva, or semen of infected animals [6]. LSD can vary from mild to severe form based on the virus’s strain and cattle breed; however, younger cattle are more susceptible and may develop the characteristic lesions within 24-48 h [7]. The most apparent clinical symptoms include fever, nasal and eye discharges, fall in milk yield, multiple nodules mainly under the skin of most body parts, and lymphadenopathy [8]. Nodules can develop in digestive and respiratory tracts to interfere with the normal functions of affected parts and predisposing for secondary bacterial infections as abomasitis and pneumonia. Furthermore, animals suffering severe may show signs of lameness, mastitis, abortions and sterility in cows and bulls [9].

In endemic countries, economic losses are depending on the rates of morbidities and mortalities as there is no specific antiviral treatment available for infected cattle [10]. However, prevention of infection and protection of susceptible animals can be made by vaccination; whereas, supportive treatment for infected ones can effectively reduce the effects of disease [11]. At the field, presumptive diagnosis depends mainly on the typical clinical signs and postmortem examination; whereas, at the laboratory, a combination of histopathological findings with the confirmatory techniques (electron microscopy, serology, and molecular assays, and viral isolation) can provide a definitive detection for LSDV [3,11,12]. Polymerase chain reaction (PCR) is one of the most useful molecular tests applied routinely to identify and confirm *Capripoxvirus* with high sensitivity and specificity [13].

In Iraq, several outbreaks were reported at different provinces throughout the past 6 years despite using of sheep poxvirus vaccine for controlling and prevention. The present study was designed to confirm LSD infection among cattle having skin lesions and suspected previously to be infected with the disease based on clinical and postmortem findings using the molecular assay and histopathology.

## Materials and Methods

### Ethical approval

The study was performed under the regulation of Veterinary Medicine/Wasit University, and the animal work was approved by the scientific and ethical committee of Veterinary Medicine/Wasit University.

### Samples and data collection

At many rural and subrural areas as well as abattoirs located in Baghdad Province/Iraq, a total of 71 cattle that diagnosed clinically to be infected acutely or chronically with LSD were subjected to the present study from October 2018 to March 2019. Pre-slaughter, data related to age, gender, and area, with measurement of temperature, pulse and respiratory rates, and level of milk production were reported. Clinical examination of the skin, lymph nodes, nose, eye, and limbs was also carried. Post-slaughter, postmortem examination was performed to detect the presence of nodular lesions in upper parts of the digestive tract, rumen, upper parts of respiratory tracts, and lungs. In addition, skin biopsies were collected immediately after slaughter, sectioned, and kept within two plastic containers; one contains 10% buffered neutral formalin solution as fixative and preservative and kept at room temperature until to be used for histopathology; and the other was saved frozen at −40°C until to be used for DNA extraction and molecular PCR assay.

### Laboratory diagnostic methods

#### Molecular PCR

DNA extraction

In this study, protocol B of G-spin kit (iNtRON, South Korea) was used to extract DNA from nodular skin lesions (lump). According to manufacturer’s instruction, a total of 25 mg of homogenized tissue sample was transferred into 1.5 ml Eppendorf tube with adding 200 µl of Buffer CL, 20 µl proteinase K, and 5 µl RNase. The tube content incubated at 56°C for 15 min, and 200 µl of Buffer BL was added, mixed, incubated at 70°C for 5 min, and centrifuged at 13,000 rpm for 5 min with removing of the up-lysed tissue particles. A 400 µl of supernatant was transferred into a new Eppendorf tube with adding a 200 µl of absolute ethanol, mixed gently, centrifuged at 13,000 for 1 min, and then transferred to spin column. A 700 µl of Buffer WA was added to spin column, centrifuged at 13,000 rpm for 1 min, discarding the infiltrate and centrifuged again at 13,000 rpm for 1 min with discarding the infiltrate. A 50 µl of Buffer CE was added to spin column that placed into new numbered Eppendorf tube, incubated for 1 min at room temperature, and centrifuged for 1 min at 13,000 rpm. Finally, sample infiltrate was kept frozen at −40°C.

DNA purification

The purity and concentration of extracted DNA were measured using Nanodrop spectrophotometer (Thermo Scientific, UK).

PCR reaction

PCR master mix was prepared by a ready to use PCR PreMix kit (BIONEER, South Korea) and performed at a final volume of 20 µl containing 5 µl DNA template, 1 µl of each primer (F and R), and 13 µl nuclease-free water. For PCR amplification, two sets of primers were used in this study: The first, *P32-F* (5´-CGCGAAATTTCAGATGTAGTTCCA-3´) and *P32-R* (5´-TGAGCCATCC ATTTTCCAACTC-3´) to amplify a 752 bp genomic fragment which specific to *P32* antigen gene [12] and the second, *DW-TKF* (5´-GCCGATAACATATATAGACCC-3´) and *OP49-R* (5´-GTGCTATCTAGTGCAGCTA T-3´) to amplify a 434 bp genomic fragment from all CaPVs, which specific to thymidine kinase (*TK*) gene [14]. PCR reactions were performed using T100 Thermal Cycler (Bio-Rad, USA) under the optimized conditions for each primer as following:

**Table:** 

Steps	Condition of the first primer			Condition of the second primer		
Initial denaturation	95°C	1 M	1 C	95°C	90 Ss	1 C
Denaturation	95°C	30 Ss	30 Cs	95°C	45 Ss	35 Cs
Annealing	58°C	30 Ss		56°C	45 Ss	
Extension	72°C	30 Ss		72°C	60 Ss	
Final extension	72°C	5 Ms	1 C	72°C	7 Ms	1 C

M: Minute, S: Second, C: Cycle, °C: Celsius

PCR analysis

The resultant PCR products were examined by electrophoresis in agarose gel (1.5%) and visualized under ultraviolet illumination after stained with ethidium bromide.

### Histopathology

#### Tissues processing

During the first 24 h of sample collection, skin specimens were dehydrated in gradual ethanol (70-100%), cleared by xylol, infiltrated, and embedded within the paraffin wax. The blocks of tissues were sectioned to the 5 µ thickness and mounted on microscopic slides.

#### Tissue staining

The mounted slides were deparaffinized with ethanol, stained with hematoxylin, washed, immersed with ethanol, stained with eosin, dehydrated with ethanol, cleared by xylol, and finally covered with a coverslip using of Canada balsam.

#### Tissue examination

The stained slides were tested using a light microscope (MEIJI, Japan) at 10×.

### Statistical analysis

All obtained results and collected data were introduced, arranged, and analyzed using Microsoft Office Excel 2016 (v16.0) and IBM^®^ SPSS (v23) (IBM Corporation, USA). Chi-square and t-test were applied to detect the significant differences at p<0.05 [15,16].

## Results

**Figure-1:**
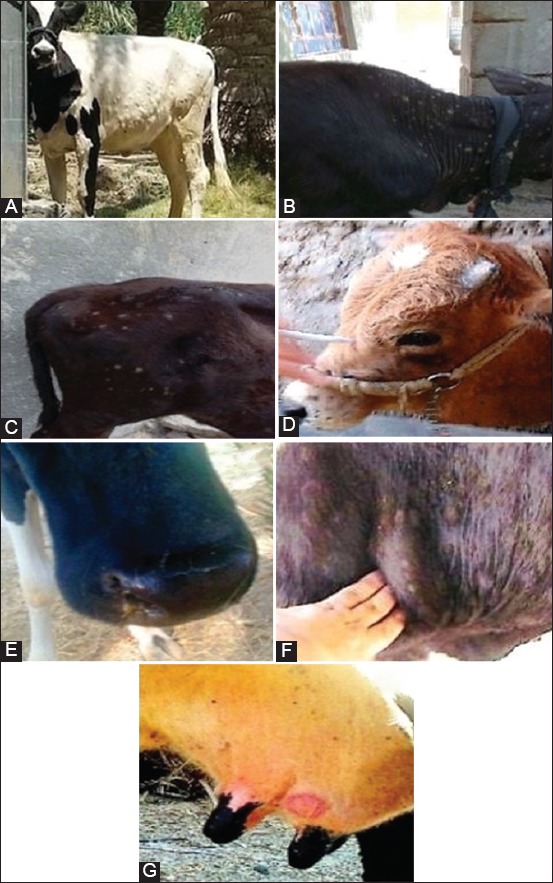
(A-G) Skin lesions in different body areas of cattle. Several skin nodules that varied in diameter, shape, and appearance were observed in various regions of the cattle.

Of 71 cattle subjected to the present study, the pre-slaughter clinical examination showed that there were significant increases in temperature (39.53-41.08°C [39.7°C±0.74]), pulse (53-107 [96.42±3.51]/min), and respiratory (27-41 [33.54±0.63]/min) rates. Multiple skin nodules varied in diameter, shape, and appearance were observed among all study animals (Figure-1). These lesions were ranged from the firm and hard to the moist and from necrotic to slough. The lesions were detected in various skin regions, particularly in the abdomen, flank, and thigh (Figure-1A), the skin of the neck (Figure-1B), in the rump and thurl (Figure-1C), muzzle (Figure-1D and E), near supramammary and/or precrural lymph nodes (Figure 1F), and on skin of the udder (Figure-1G). Other symptoms include enlargement of lymph nodes (prescapular, supramammary, and prefemoral), lacrimation, mucopurulent nasal discharge, salivation, edema in limbs and head among severe infected cases, and marked fall in milk production.

**Figure-2:**
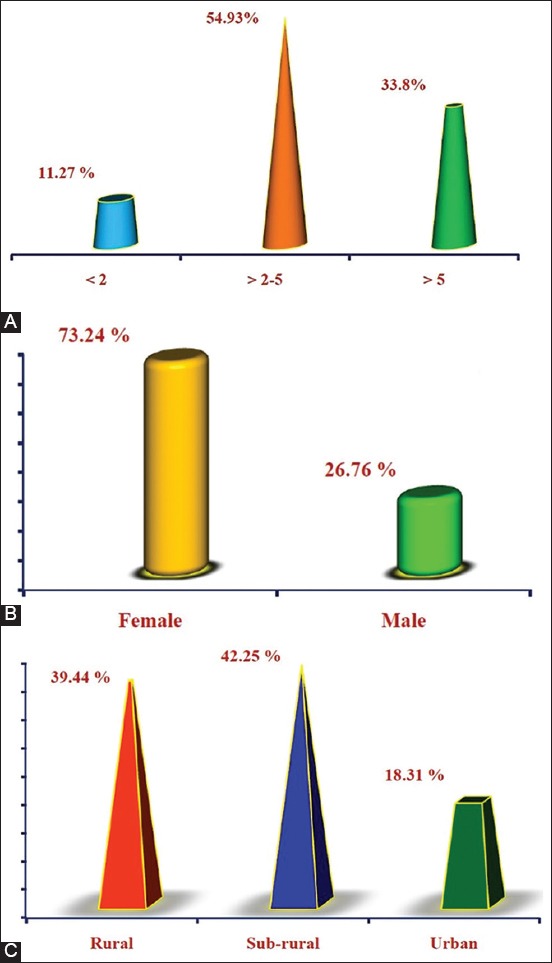
Association of the disease prevalence to epidemiological risk factors. The association of the disease to risk factors including (A) age, (B) gender, and (C) areas was assessed to identify the rate of disease prevalence in response to age, gender, and geographic area.

**Figure-3:**
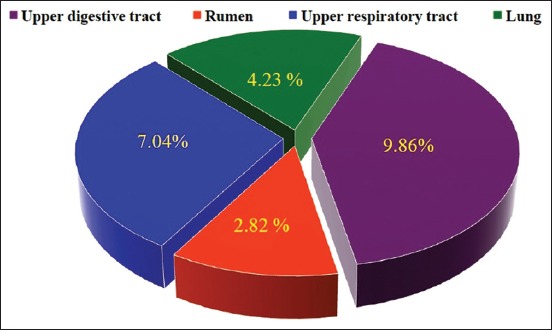
Postmortem detection of nodular lesions in digestive and respiratory tracts. Distribution of nodular lesions in digestive and respiratory tracts was investigated in slaughter cattle.

**Figure-4:**
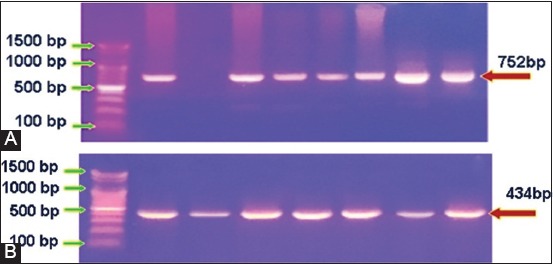
Polymerase chain reaction analysis of the positive samples. (A) Gel electrophoresis of P32 gene of positive samples amplified at 752 bp. (B) Gel electrophoresis of thymidine kinase of positive samples amplified at 434 bp. The displayed images are represented 64 and 43 samples, respectively.

An association of the disease to risk factors including age, gender, and areas was investigated and the result showed significant elevation of LSD prevalence among the group of >2-5 years (39/71 [54.93%]) rather than >5 years (24/71 [33.8%]) and <2 years (8/71 [11.27%]) group, respectively (Figure-2A). Furthermore, increased prevalence was detected in females (52/71 [73.24%]) more than males (19/71 [26.76%]) (Figure-2B). Moreover, the subrural (30/71 [42.25%]) and rural (28/71 [39.44%]) areas were reported significant increases of LSD prevalence compared to urban (13/71 [18.31%]) areas (Figure-2C). After slaughter, postmortem examination found that their nodular lesions were seen in upper parts of digestive tract (7/71 [9.86%]), rumen (2/71 [2.82%]), upper respiratory tracts (5/71 [7.04%]), and lung (3/71 [4.23%]) (Figure-3). Overall, the results of PCR using two sets of primers that targeted *P32* and *TK* antigenic genes, respectively (Figure-4).

**Figure-5:**
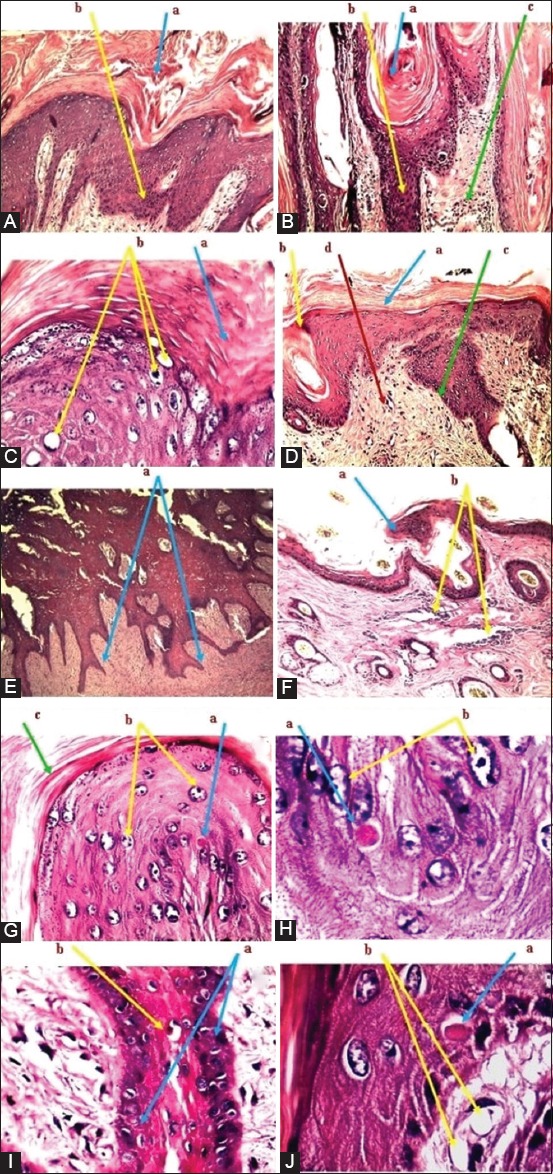
Histopathological analysis of skin nodular lesions. (A): (a) Hyperkeratosis in the epidermis. (b) Hyperplasia of stratum basale. (B): (a) Hyperkeratosis. (b) Hyperplasia. (c) Inflammatory cell infiltration. (C): (a) Hyperkeratosis. (b) Vacuolation with swelling of granulocytes. (D): (a) Hyperkeratosis. (b) Acanthosis. (c) Hyperplasia (d) Inflammatory cell infiltration. (E): (a) Hyperplasia toward dermis. (F): (a) Acanthosis. (b) Inflammatory cell infiltration in dermis. (G): (a) Intracytoplasmic inclusion bodies. (b) Vacuolation of granulocytes. (c) Hyperkeratosis. (H): (a) Eosinophilic inclusion bodies. (b) Vacuolation of granulocytes. (I): (a) Eosinophilic inclusion bodies. (b) The proliferation of basal cells in the epidermis. (J): (a) Eosinophilic inclusion bodies. (b) Vacuolation of granulocytes. The tissue stained with hematoxylin and eosin stain and examined under a light microscope and imaged at 10×. All displayed images are represented all detected positive samples.

Histopathological analysis of nodular skin lesion showed that there were edema, hyperemia, acanthosis, severe hydropic degeneration, and hyperkeratosis in the epidermis (Figure-5A-E), whereas in the dermis, mononuclear cell infiltration, inclusion bodies, and vasculitis were seen (Figure-5F-J).

## Discussion

LSD is an exhausted viral disease affecting many countries, particularly in Africa and Asia resulting economically in great losses due to the high rates of morbidity, chronic debilitation in diseased cattle, abortion, severe reduction in milk production, weak growth, damage to hides, and temporary or permanent sterility. Besides, it is considered one of the transboundary animal diseases for its significant impact on trade and food security and the ability to spread to other countries [17]. The real danger of the disease lies in the fact that it has continued to spread and to extend in its range to include new areas, countries, and fields [18]. In the current study, the case history and clinical examination data are mostly correlated with LSD. These findings were similar to those previously described by Awadin *et al*. [19] Body *et al*. [20] Ahmed and Dessouki, [21]. In Iraq, many outbreaks have been reported during the past 6 years, which varied in their severity and incidence; nonetheless, it is unclear how the disease is maintained during interepidemic periods. It thought that LSDV could preserve by either the inapparent infections cycled in cattle or old lesions or the role of wildlife animals in pathogenesis [22]. However, it is claimed that the very young calves, lactating cows, and animals suffering from malnutrition were developed generally the most severe infections probably due to an impairment of cellular immunity [18]. Besides, the high ambient temperature coupled with farming practices to produce high milk might be deemed for stressing of these animals and contribute to the severity of the disease [23].

Association of infection to the epidemiological risk factors (age, gender, and area) of study’s cattle found that LSD was more prevalent among groups of >2-5 years, females, and subrural as well as rural areas; and these findings were similar, respectively, to that reported by Ahmed and Dessouki [21] Ayelet *et al*. [24], Abera *et al*. [25]. The lowest attack of LSD in animals of <2 years might be indicative of the low-frequency exposure, lower susceptibility to biting flies, and prevailing passive maternal immunity [26]. The higher cumulative incidence of LSD in females than males might be attributed to stress factors of fatigue and exhaustion rather than to a biological cause, low numbers of males found in the field compared to females, and low exposure of bulls to biting insects. The prevalence of LSD among subrural and rural areas could occur due to more enormous proportions of farm animals cultivated in these areas, non-adoption of control measures, and favorable environmental conditions.

Differences in the propagation of nodules in upper digestive and respiratory tracts observed in this study with the presence of rumen and lung lesions were agreed with El-Kenawy and El-Tholoth [27]. The mucous membrane of upper digestive and respiratory tracts is often developed multiple, discrete ulcerative lesions irrespective of the numbers of skin nodules resulting in difficulties in swallowing and severe asphyxia and dyspnea. In rare cases, nodular lesions might find in the internal organ as rumen, lung, kidney, udder, uterus, and testes [28]. These findings might be attributed to the fact that LSDV infections are systemic, and the virus disseminates to various tissues through a leukocyte-associated viremia [29]. As well as, the presence of additional stresses on cattle in a field may exacerbate the disease [30]. The exact pathogenesis for the development of lesions associated with LSD in multiple organ systems has not been well understood. However, it suggested that the tissue tropism and shedding of LSD might show some similarities to capripoxvirus infections in sheep and goats [31].

PCR examination for extracted DNA from skin lesions of study’s cattle using PCR assay revealed that 90.14% and 60.56% were positive samples for primers targeted *P32* and *TK* genes, respectively. *P32* protein is a highly antigenic structural protein of all strains of CaPVs, which play an essential role in pathogenicity and diagnosis of LSDV to be used widely in serological and molecular detection of the virus [32]. It reported that the *P32* gene is the better candidate gene for differentiating between vaccine strains from field isolates and for illustrating genetic variations between LSDVs [33,34]. Besides, it indicated that the possibility of this gene for confirmation up to 100% of representative infected cattle among skin biopsies provided high sensitivity and specificity in the detection of LSDV [35]. Furthermore, biopsies of the scabs or skin lesions are more suitable, distinguishable and represented sample for diagnosis of LSD as they substantial, progress to sit-fasts and contain abundant DNA of LSDV [36]. Negative samples could be due to either of the complete absence of LSDV in a specimen or deficient level of virus that presents in tissue sample below the sensitivity of the assay [19,35].

Although histopathological findings of LSDV varied in their characteristics considerably depending on the developmental stage, they can provide a basis for diagnosis [6,37]. In the current study, histopathology of skin nodules showed that most cutaneous lesions were extended throughout all layers of skin and illustrated a profuse hyperkeratosis, acanthosis with marked vacuolation, cloudy swelling of granulocytes in stratum granulosum, and downward hyperplasia of stratum basale. Moreover, infiltration of inflammatory cells among the layers of dermis and thickening in walls of hair follicles was observed. Eosinophilic intracytoplasmic LSD viral inclusion bodies were detected microscopically within the cytoplasm of granulocytes in stratum basale, and some lesions appeared as papillary projections due to the proliferation of cells in stratum basale. In acute stages, LSD can comprise the granulomatous reactions in dermis and hypodermis, which extended to surrounding tissues, with the existence of vasculitis, lymphangitis, and thrombosis, and infarction that resulted in necrosis and edema. The presence of intracytoplasmic eosinophilic inclusions can be considered as a hallmark for acute and subacute stages of LSD lesions [38]. As detected by Babiuk *et al*. [30], it found that there was a significant variation in microvesicles, hyperkeratosis or acanthosis, presence of eosinophils, and the prominent vascular changes among skin lesions of naturally infected cattle. The variation could be due to the differences in disease progression stage at which the samples were collected or virus strain differences [18]. In the field, numerous skin diseases can persist, which confusing the diagnosis of LSD as pseudo-LSD that could be differentiated clinically by lack of systemic signs and histologically as the lesions will involve only the epidermis and leave scabs after sloughing [39]. Urticaria, tick bites, and insect bites/stings may confuse with LSD but can be differentiated by the absence of eosinophils and presence of deep vasculitis with intracytoplasmic inclusion bodies in keratinocytes and epithelium of hair follicles [40].

## Conclusion

In Iraq, despite vaccination programs applied since the first outbreak in 2013, LSDV remains large in persistence and distribution among most areas of the country, resulting in apparent morbidities and mortalities. In our knowledge, the present study was the first one that dealt with LSD-related epidemiological risk factors and postmortem examination. To support clinical signs, effective control of LSD requires an accurate and rapid laboratory diagnostic method like PCR assay, which considered the best available test of choice for the identification of the disease.

## Authors’ Contributions

HAJG and MNKA were responsible for clinical examination and samples collection, SJJA was responsible for histopathology examination, and HHHA was responsible for molecular examination. All authors contributed in writing this manuscript. All authors read and approved the final manuscript.
